# A case report of a prolonged decrease in tacrolimus clearance due to co-administration of nirmatrelvir/ritonavir in a lung transplant recipient receiving itraconazole prophylaxis

**DOI:** 10.1186/s40780-023-00280-3

**Published:** 2023-04-01

**Authors:** Ayumi Tsuzawa, Yoshiki Katada, Keisuke Umemura, Mitsuhiro Sugimoto, Asami Nishikawa, Yu-ki Sato, Yuko Yoshida, Noriaki Kitada, Atsushi Yonezawa, Daisuke Nakajima, Hiroshi Date, Tomohiro Terada

**Affiliations:** 1 Department of Clinical Pharmacology and Therapeutics, Kyoto University Hospital, 54 Kawahara-Cho, Shogoin, Sakyo-Ku, Kyoto, 606-8507 Japan; 2 Department of Thoracic Surgery, Kyoto University Graduate School of Medicine, 54 Kawahara-Cho, Shogoin, Sakyo-Ku, Kyoto, 606-8507 Japan

**Keywords:** Tacrolimus, Nirmatrelvir/ritonavir, Itraconazole, Novel coronavirus disease 2019, Drug-drug interaction, Lung transplantation, CYP3A

## Abstract

**Background:**

Drug-drug interaction management is complex. Nirmatrelvir/ritonavir is a potent cytochrome P450 (CYP) 3A inhibitor and influences pharmacokinetics of co-administered drugs. Although there are several reports about drug-drug interactions of nirmatrelvir/ritonavir, an influence of a concomitant use of nirmatrelvir/ritonavir and another potent CYP3A inhibitor on tacrolimus remains unclear. Here, we experienced a lung transplant patient with the novel coronavirus disease 2019 (COVID-19). In this patient, nirmatrelvir/ritonavir was administered, and the inhibitory effect of itraconazole on CYP3A was prolonged.

**Case presentation:**

We present a case in forties who had undergone lung transplantation. He was administered itraconazole and tacrolimus 1.0 mg/d, with a trough value of 8–12 ng/mL. The patient contracted the COVID-19, and a nirmatrelvir/ritonavir treatment was initiated. During the antiviral treatment, tacrolimus administration was discontinued for 5 d. Tacrolimus was resumed at 1.0 mg/d after completion of the nirmatrelvir/ritonavir treatment, but the trough value after 7 d was high at 31.6 ng/mL. Subsequently, the patient was placed on another 36-h tacrolimus discontinuation, but the trough value decreased to only 16.0 ng/mL.

**Conclusions:**

Co-administration of ritonavir caused a prolonged decrease in tacrolimus clearance through its inhibitory effects on CYP3A in a patient taking itraconazole. Management of drug-drug interaction by pharmacists can be important for patients with multiple medications.

## Background

Patients who have undergone solid organ transplants require long-term immunosuppressive therapy. Consequently, they are at a high risk of acquiring severe illness from the novel coronavirus disease 2019 (COVID-19) [1]. Nirmatrelvir/ritonavir (NMV/RTV) has been useful for patients with mild to moderate infection and severe illness risk factors [2]. NMV/RTV exerts a strong cytochrome P450 (CYP) 3A inhibitory effect to maintain an effective concentration of NMV. Hence, paying attention to drug interactions with concomitant medications is necessary [3]. In particular, many solid-organ transplant patients are on tacrolimus (TAC) therapy, a substrate drug for CYP3A4/5. It is essential to predict the effect on blood TAC concentration and manage drug interactions when both drugs are administered.

Mertz et al. reported that in renal transplant patients with human immunodeficiency virus (HIV)-associated nephropathy, blood concentration of TAC can be controlled during concomitant use of darunavir/RTV by reducing TAC to 3.5% of the baseline level [4]. Rose et al. also reported that the concomitant use of NMV/RTV in a pancreatic kidney transplant patient taking TAC resulted in acute renal dysfunction with a blood TAC concentration of ≥ 60 ng/mL [5]. It is recommended to discontinue or reduce TAC dosage and monitor blood TAC levels during NMV/RTV treatments [6]. However, lung transplant patients have a higher risk of developing aspergillus infection than solid-organ transplant patients. Itraconazole (ITCZ) or voriconazole, potent CYP3A inhibitors, are important after lung transplantation [7]. Coadministration of ITCZ increases the blood concentration of TAC. Therefore, dose adjustment of TAC based on blood concentration monitoring is necessary [8]. Interactions between multiple potent CYP3A inhibitors and TAC must be considered during concomitant NMV/RTV administration in lung transplant patients.

There are many cases wherein multiple drugs inhibit CYP activity. Although there are several reports about drug-drug interactions of NMV/RTV, an influence of a concomitant use of NMV/RTV and another potent CYP3A inhibitor on tacrolimus remains unclear. Here, we experienced a case in which NMV/RTV was administered to a lung transplant patient with COVID-19, and the CYP3A inhibitory effect of ITCZ was prolonged, which reduced TAC clearance.

## Case presentation

The patient was a man in forties. He had no particular allergies or history of side effects. He underwent bilateral living-donor lung transplantation at the Department of Thoracic Surgery of Kyoto University Hospital (hereafter referred to as this hospital) for idiopathic pulmonary fibrosis and interstitial pneumonia 8 years prior to 20XX (20XX–8). He had been taking TAC 1.0 mg/day and ITCZ 200 mg/day. The trough TAC level during the past year was well-controlled at approximately 10 ng/mL (target trough value: 8–12 ng/mL), and the troughs of ITCZ and its active metabolite hydroxy ITCZ were confirmed as 455 ng/mL and 914 ng/mL, respectively, when ITCZ was started in 20XX–8. His regular medications comprised TAC 0.5 mg (one capsule each, BID, at 9:00/21:00), mycophenolate mofetil 250 mg (one capsule each, BID, after breakfast and dinner), prednisolone 5 mg (2.5 tablets, OD, after breakfast on alternative days), sulfamethoxazole 400 mg and trimethoprim 80 mg (one tablet each, OD, after breakfast on Mondays, Wednesdays, and Fridays), itraconazole 100 mg (one tablet each, BID, immediately after breakfast and dinner), verapamil 40 mg (one tablet each, BID, after breakfast and dinner), edoxaban 30 mg (one tablet, OD, after breakfast), polaprezinc 75 mg (one tablet each, BID, after breakfast and before sleep), and esomeprazole 20 mg (one capsule, OD, after breakfast).

**Fig. 1:**
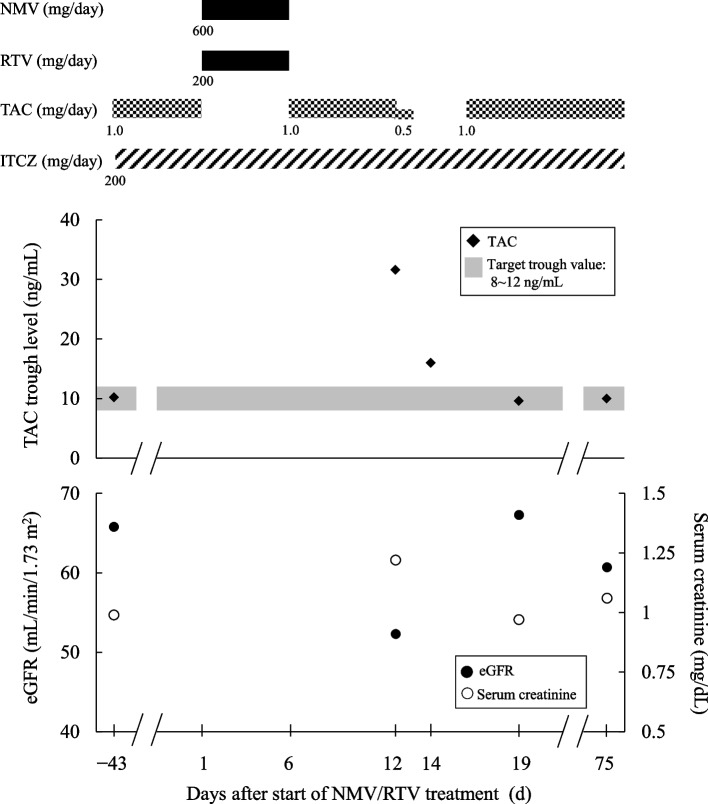
The clinical course of the present patient. The doses of NMV/RTV, TAC and ITCZ, TAC trough levels, and laboratory parameters are shown. NMV: nirmatrelvir, RTV: ritonavir, TAC: tacrolimus, ITCZ: itraconazole, eGFR: estimated glomerular filtration rate

The patient sought medical attention at his nearby clinic in Y, 20XX after experiencing fever and breathing discomfort and was diagnosed with COVID-19 (Day 1). On the same day, the clinic prescribed NMV/RTV (NMV 300 mg, RTV 100 mg, BID for 5 d), and decided for the patient to rest and be treated at home. The patient was placed on a TAC discontinuation during the 5 days of NMV/RTV treatments. TAC was resumed at the pre-NMV/RTV treatment dose of 1.0 mg/day after the completion of NMV/RTV treatments (Day 6). The trough TAC level 43 days prior to taking NMV/RTV (Day -43) was 10.2 ng/mL. On Day 12, after completion of the at-home rest and treatment, the patient visited the Department of Thoracic Surgery of this hospital; the trough TAC level was high at 31.6 ng/mL. Even though he did not present with headache or elevated blood pressure, his serum creatinine level increased from 0.99 mg/dL (Day -43) to 1.22 mg/dL. His estimated glomerular filtration rate (eGFR) decreased from 65.8 mL/min/1.73 m^2^ to 52.3 mL/min/1.73 m^2^. Laboratory parameters of hepatic function, electrolyte, and hematocrit level were all normal. The patient was placed on a 36-h TAC discontinuation from Day 12 21:00 dose to Day 13 (full day). The blood TAC level was measured again on Day 14. The TAC trough level was 16.0 ng/mL, above the upper limit target (12 ng/mL). Therefore, he was placed again on a TAC discontinuation for 48 h from Day 14 to Day 15. The TAC treatment was resumed from Day 16 at 1.0 mg/d, the same dose as before starting NMV/RTV treatments. The TAC trough level on Day 19, the fourth day after resuming TAC, was 9.6 ng/mL, within the target range. The serum creatinine level and eGFR were 0.97 mg/dL and 67.3 mL/min/1.73 m^2^, respectively. Aside from TAC, no other adjustments were required for the doses of the other medications before and after NMV/RTV treatments. The Fig. 1 shows the clinical course of the doses of NMV/RTV, TAC, and ITCZ, as well as the TAC trough levels and various other laboratory parameters.

## Discussion and conclusions

Administration of TAC and ITCZ is necessary after lung transplantation. We should pay an attention to the complex drug-drug interactions with concomitant drugs in these patients. In the present case, a patient on ITCZ therapy received NMV/RTV treatments, and an elevated TAC trough level was confirmed 7 d after the end of NMV/RTV treatment. Despite several days of TAC discontinuation, the drug interactions persisted. Blood concentration of TAC was difficult to manage due to complex concomitant drug interactions from the administration of a potent CYP3A inhibitor in addition to RTV.

The NMV/RTV package insert (PaxlovidPACK®, Pfizer Japan Inc., revised in September 2022 [4th edition]) cautions regarding drug interactions with TAC. However, no clear standards for discontinuation or dose reduction are recommended. Berar et al. reported that concomitant use of NMV/RTV reduced TAC clearance to approximately 3.9% in renal transplant patients. Therefore, the authors recommended that TAC be withdrawn while taking NMV/RTV [9]. In the previous reports on NMV/RTV treatment for solid-organ transplant recipients, TAC was stopped during NMV/RTV treatment and restarted on the day after completion of treatment [10]. The TAC trough levels were maintained within therapeutic ranges. In this case, as well, TAC treatments were suspended during the NMV/RTV treatment period, and oral administration of TAC was resumed the day after NMV/RTV administration was completed at the same dose as before the onset of COVID-19. However, an increase in TAC trough levels was observed despite implementing TAC management methods. Stader et al. reported that the CYP3A inhibitory effect after discontinuing RTV decreased by 46–61% one day after discontinuation and by 70–90% two to five days after discontinuation. However, it took 3 weeks for the inhibitory effect to subside completely [11]. In addition, Mecadon et al. reported that when TAC was resumed within 24 h after the end of NMV/RTV treatment, the CYP3A inhibitory effect of RTV was prolonged. As a result, the upper limit of the TAC target blood concentration was exceeded [12]. It was recently reported that TAC concentration did not decrease within 48 h after completing NMV/RTV [13]. Dewey KW et al. have reported restarting TAC at 25% of the original dose 48 h after completing NMV/RTV treatment [14]. The appropriate dose of TAC to restart after completing NMV/RTV treatment may depend on the patient's target blood concentration of TAC. Therefore, when resuming TAC after completing NMV/RTV, the TAC blood concentration should be determined before resuming treatment. The TAC dose and timing should be considered based on the results.

In this case, a 36-h TAC discontinuation reduced blood TAC concentration from 31.6 ng/mL to 16.0 ng/mL. The estimated half-life of TAC on Day 12 was approximately 36 h, whereas the reported half-life of TAC is approximately 12–19 h [15, 16], suggesting that the half-life had been extended in our case. ITCZ, which this patient was administering concomitantly, has a CYP3A inhibitory effect and, therefore, is expected to extend the half-life of TAC. However, since the blood concentration/dose ratio of TAC was higher than that before NMV/RTV use, it suggested that TAC clearance was further decreased, and the half-life was prolonged on Day 12. ITCZ is also metabolized by CYP3A, a metabolic pathway similar to that of RTV [17]. Crommentuyn et al. published a case report, wherein the concomitant use of anti-HIV drugs including RTV in a patient administering ITCZ increased blood levels of ITCZ [18]. The half-life of ITCZ has been extended from 16 h to > 160 h. This suggests that RTV causes an excessive increase in blood ITCZ levels, and it may take several days to achieve the original blood ITCZ levels. The IC_50_ value of free ITCZ on CYP3A4 was 32.6 nM (23.0 ng/mL) [19]. The serum concentration of ITCZ in this patient was 645 nM (455 ng/mL). Because the protein binding ratio of ITCZ is reported to be 99.8% on the package insert, CYP3A4 activity cannot be completely suppressed only by ITCZ. In addition, Ohno et al. reported that the apparent inhibition ratio of ITCZ on CYP3A4 was 95% [20]. Therefore, further inhibition of CYP3A4 was suggested to further reduce TAC clearance. Although we had not determined the blood ITCZ and RTV levels in the present case, we believe that co-administration of RTV delayed ITCZ clearance, leading to prolongation of the potentiated CYP3A inhibitory effect of ITCZ, resulting in reduced TAC clearance even after 7 d of RTV discontinuation. On the other hand, RTV was also metabolized by CYP3A [21]. ITCZ may also decrease RTV clearance and potentiate an inhibitory effect of RTV on CYP3A4. The other concomitant medications had little effects on TAC clearance because they do not have inhibitory or inducing effects on the enzymes and transporters that are involved in the pharmacokinetics of TAC. When NMV/RTV is started for patients who concomitantly take strong CYP3A inhibitors, e.g. ITCZ, clinicians should consider dose reduction or discontinuation of these CYP3A inhibitors as well as TAC.

Although headaches and elevated blood pressure are adverse reactions expected in response to elevated blood TAC concentrations, these did not occur in the current patient. Renal dysfunction was noted on Day 12. Although the influence of COVID-19 cannot be ruled out, we concluded that renal dysfunction was the result of elevated blood TAC concentration for three reasons: 1) clear temporal relationship between the increase in blood TAC concentration and renal dysfunction; 2) TAC trough level reaching a toxic range; and 3) serum creatinine level and eGFR improved with decreasing TAC trough levels. The “Guidelines for Management of Drug Interactions of Paxlovid (Nirmatrelvir/Ritonavir)—Ver.1.1” published by the *Journal of Pharmaceutical Health Care and Sciences* recommend to “Avoid NMV/RTV use in patients taking TAC unless blood TAC levels can be closely monitored” [22]. To administer TAC more safely, therapeutic agents other than NMV/RTV should be considered in the treatment of COVID-19. This patient did not develop side effects other than the elevated TAC levels. However, side effects such as confusion, somnolence, and diarrhea caused by the elevated blood ITCZ concentration after a concomitant use of RTV and ITCZ have been reported [23]. Therefore, it is necessary to reinforce the monitoring of side effects for not only TAC but also for ITCZ during NMV/RTV treatments.

Patients undergoing solid-organ transplantation require a long-term immunosuppressant therapy. Hence, they are at high risk of severe COVID-19. During the infection, appropriate antiviral drugs must be administered. In general, the CYP3A inhibitory effect of RTV is considered to fade after 3–5 d of treatment discontinuation. However, our experience with this case suggests that we should pay an attention to drug-drug interaction more than 5 days after NMV/RTV discontinuation in patients receiving a strong CYP3A inhibitor. Accordingly, when NMV/RTV is administered to patients receiving TAC and a strong CYP3A inhibitor, careful blood TAC concentration monitoring is required after 3–5 d of NMV/RTV termination.

## Data Availability

Data used in this case report will not be shared owing to the risk of identifying an individual.

## Abbreviations

COVID-19: Coronavirus infection
NMV/ RTV: Nirmatrelvir/ritonavir
CYP: Cytochrome P450
TAC: Tacrolimus
HIV: Human immunodeficiency virus
ITCZ: Itraconazole
eGFR: Estimated glomerular filtration rate

## References

[1] Pereira MR, Mohan S, Cohen DJ, Husain SA, Dube GK, Ratner LE (2020). COVID-19 in solid organ transplant recipients: initial report from the US epicenter. Am J Transplant.

[2] Hammond J, Leister-Tebbe H, Gardner A, Abreu P, Bao W, Wisemandle W (2022). Oral nirmatrelvir for high-risk, nonhospitalized adults with covid-19. N Engl J Med.

[3] Marzolini C, Kuritzkes DR, Marra F, Boyle A, Gibbons S, Flexner C (2022). Prescribing nirmatrelvir-ritonavir: how to recognize and manage drug-drug interactions. Ann Intern Med.

[4] Mertz D, Battegay M, Marzolini C, Mayr M (2009). Drug-drug interaction in a kidney transplant recipient receiving HIV salvage therapy and tacrolimus. Am J Kidney Dis.

[5] Rose DT, Gandhi SM, Bedard RA, Mondy KE, Chu AL, Gamble KC (2022). Supratherapeutic tacrolimus concentrations with nirmatrelvir/ritonavir in solid organ transplant recipients requiring hospitalization: a case series using rifampin for reversal. Open Forum Infect Dis.

[6] Fishbane S, Hirsch JS, Nair V (2022). Special considerations for paxlovid treatment among transplant recipients with SARS-CoV-2 infection. Am J Kidney Dis.

[7] Gavaldà J, Meije Y, Fortún J, Roilides E, Saliba F, Lortholary O (2014). Invasive fungal infections in solid organ transplant recipients. Clin Microbiol Infect.

[8] Kramer MR, Amital A, Fuks L, Shitrit D (2011). Voriconazole and itraconazole in lung transplant recipients receiving tacrolimus (FK 506): efficacy and drug interaction. Clin Transplant.

[9] Berar Yanay N, Bogner I, Saker K, Tannous E (2022). Paxlovid-tacrolimus drug-drug interaction in a 23-year-old female kidney transplant patient with COVID-19. Clin Drug Investig.

[10] Salerno DM, Jennings DL, Lange NW, Kovac D, Shertel T, Chen JK (2022). Early clinical experience with nirmatrelvir/ritonavir for the treatment of COVID-19 in solid organ transplant recipients. Am J Transplant.

[11] Stader F, Khoo S, Stoeckle M, Back D, Hirsch HH, Battegay M (2020). Stopping lopinavir/ritonavir in COVID-19 patients: duration of the drug interacting effect. J Antimicrob Chemother.

[12] Mecadon K, Arvanitis P, Farmakiotis D, Rogers R (2022). Single-center experience with nirmatrelvir/ritonavir in kidney transplant recipients on tacrolimus maintenance immunosuppression. Clin Transplant.

[13] Léonard G, Camille T, Bénédicte F, Cécile V, Marie-Clémence V, Florian L (2023). First experience of optimization of tacrolimus therapeutic drug monitoring in a patient c otreated with nirmatrelvir/ritonavir: How microsampling approach changes everything. Transplantation.

[14] Dewey KW, Yen B, Lazo J, Seijo L, Jariwala R, Shah R, et al. Nirmatrelvir/ritonavir use with tacrolimus in lung transplant recipients: a single-center case series. Transplantation. 2022; Online ahead of print. 10.1097/TP.0000000000004394. 10.1097/TP.0000000000004394 PMC10125013 36525555

[15] Sikma MA, van Maarseveen EM, van de Graaf EA, Kirkels JH, Verhaar MC, Donker DW (2015). Pharmacokinetics and toxicity of tacrolimus early after heart and lung transplantation. Am J Transplant.

[16] Scott LJ, McKeage K, Keam SJ, Plosker GL (2003). Tacrolimus: a further update of its use in the management of organ transplantation. Drugs.

[17] MacKenzie-Wood AR, Whitfeld MJ, Ray JE (1999). Itraconazole and HIV protease inhibitors: an important interaction. Med J Aust.

[18] Crommentuyn KM, Mulder JW, Sparidans RW, Huitema AD, Schellens JH, Beijnen JH (2004). Drug-drug interaction between itraconazole and the antiretroviral drug lopinavir/ritonavir in an HIV-1-infected patient with disseminated histoplasmosis. Clin Infect Dis.

[19] Sakaeda T, Iwaki K, Kakumoto M, Nishikawa M, Niwa T, Jin JS (2005). Effect of micafungin on cytochrome P450 3A4 and multidrug resistance protein 1 activities, and its comparison with azole antifungal drugs. J Pharm Pharmacol.

[20] Ohno Y, Hisaka A, Suzuki H (2007). General framework for the quantitative prediction of CYP3A4-mediated oral drug interactions based on the AUC increase by coadministration of standard drugs. Clin Pharmacokinet.

[21] Kumar GN, Rodrigues AD, Buko AM, Denissen JF (1996). Cytochrome P450-mediated metabolism of the HIV-1 protease inhibitor ritonavir (ABT-538) in human liver microsomes. J Pharmacol Exp Ther.

[22] Japanese Society of Pharmaceutical Health Care and Sciences. Guidelines for management of drug interactions of paxlovid (nirmatrelvir/ritonavir) - Ver.1.1. 2022. https://www.jsphcs.jp/news/2022/0228-11.pdf (in Japanese). Accessed 10/2022.

[23] Le Meur L, Tantet C, Lê MP, Desselas E, Bonnal C, Lillo-Le-Louet A (2018). Serious neuropsychiatric adverse effects related to interaction between itraconazole and darunavir/ritonavir in an HIV-infected patient with cerebral histoplasmosis. J Antimicrob Chemother.

